# Isolation of Carrot Chromoplasts and Assessment of Their Carotenoid Content and Bioaccessibility

**DOI:** 10.3390/molecules30061267

**Published:** 2025-03-12

**Authors:** Ana M. Benítez-González, Lourdes Gómez-Gómez, Oussama Ahrazem, Patricia Esquivel, Carla M. Stinco, Antonio J. Meléndez-Martínez

**Affiliations:** 1 Food Colour and Quality Laboratory, Facultad de Farmacia, Universidad de Sevilla, 41012 Sevilla, Spain; abenitez@us.es (A.M.B.-G.); ajmelendez@us.es (A.J.M.-M.); 2 Instituto Botánico, Universidad de Castilla-La Mancha, 02006 Albacete, Spain; marialourdes.gomez@uclm.es (L.G.-G.); oussama.ahrazem@uclm.es (O.A.); 3 Departamento de Ciencia y Tecnología Agroforestal y Genética, Facultad de Farmacia, Universidad de Castilla-La Mancha, 02006 Albacete, Spain; 4 School of Food Technology, University of Costa Rica, San José 11501-2060, Costa Rica; patricia.esquivel@ucr.ac.cr

**Keywords:** colourless carotenoids, phytoene, phytofluene, plastids, provitamin A carotenoids

## Abstract

The bioaccessibility (fraction of compounds released from the food matrix and available for absorption) and carotenoid content of carrot chromoplasts obtained through high-speed centrifugation using sucrose gradients were assessed. Three chromoplast bands were isolated, corresponding to sucrose gradients between 15 and 30%, 30 and 40%, and 40 and 50%. Total carotenoid levels increased ~2.8-fold when comparing the fractions of the bands of the lowest and highest sucrose gradients. The carotenoid profiles of the bands were similar. Phytoene and phytofluene accounted for approximately 3 and 4%, respectively, while ζ-carotene made up about 3%. Provitamin A carotenoids comprised about 85% of the total carotenoids in the respective fractions. Lutein content varied among fractions, with 1.61% in the 15/30% band and 0.77% in the 40/50% sucrose band. Similar micellar carotenoid profiles were also observed across fractions. α-carotene and β-carotene accounted for 8% and 0.2% of the total carotenoid content, respectively, while ζ-carotene constituted 19%. Lutein content in micelles ranged from 0.5% in the highest sucrose content fractions to 3.2% in the lowest. Phytoene and phytofluene were the predominant carotenoids in micelles. They accounted for 41.7% and 28.4%, respectively, together representing 70% of all carotenoids, with no differences among fractions. Colourless carotenoids were more readily incorporated into micelles, followed by ζ-carotene, lutein, and provitamin A carotenoids.

## 1. Introduction

It is well known that carrot (*Daucus carota*) represents a significant dietary source of carotenoids, particularly β-carotene, relevant for its provitamin A activity [1]. However, carrots contain important amounts of other carotenoids, such as α-carotene, lutein, and the colourless carotenoids, phytoene and phytofluene, which are attracting increased interest in relation to health promotion and as nutricosmetics ingredients [2]. In particular, carotenoids such as phytoene and phytofluene have demonstrated emerging applications in the context of skin health and protection against UV radiation [2].

It is important to note that the carotenoid content of plant foods such as carrots is highly variable due to genetic factors, climatic and agronomic factors (type of soil, type of fertilizer used, farming techniques), maturity stage, post-harvest handling or storage conditions, among others [3,4,5].

Carotenoids accumulate in specialized plastids giving colours to many flowers, fruits, and vegetables. These are cellular organelles that have evolved to store pigments in plants. In photosynthetic tissues, carotenoids are deposited in chloroplasts, that can develop to chromoplasts during fruit ripening. Chromoplasts may also evolve from non-coloured plastids in some root vegetables. Chromoplasts exhibit diverse morphologies in their carotenoid-accumulating substructures, including globular, crystalline, membranous, fibrillar, and tubular forms [6,7,8]. Furthermore, they are surrounded by the organelle and cell membranes, as well as by the cell wall. All these factors, as well as the physical state of carotenoids and their location, affect their release from the food matrix [9,10]. In carrot roots, β-carotene is deposited as crystalline aggregates in chromoplasts. This form of accumulation may be a cellular strategy to accumulate high concentrations of these compounds. Crystallization of carotenoids may reduce their bioaccessibility, as crystalline forms are less soluble and more difficult to release [11]. The organization and physicochemical state of carotenoids within the cell are important to better understand their accumulation, stability and release, for instance during extractions or the digestion. The presence of these different forms influences the stability and release of the carotenoids during the digestive process. The carotenoid content of fruits is determined not only by the rate of biosynthesis and degradation but also by the capacity to deposit carotenoids in chromoplasts [12].

The isolation of chromoplasts represents a valuable tool for the more precise study of the content and distribution of carotenoids in plant cells. The different fractions isolated contain chromoplast fractions, different types of chromoplasts or chromoplasts at different development stages, which are separated largely according to their composition and structure, which in turn affect their density. The analysis of isolated chromoplasts allows us to gain further insight into the impact of their structure and internal organization on the release of carotenoids during digestion. This approach is useful for fine-tuning strategies that enhance the bioavailability of these compounds in foodstuffs. Different authors described methodologies for chromoplast isolation as well as their purification to obtain fractions by means of sucrose gradient and high-speed centrifugation [6,13].

The current lack of detailed comprehension of the location and organization of carotenoids within chromoplasts presents a significant challenge in better understanding their bioaccessibility. The separation of selected fractions, including chromoplasts whose characteristics facilitate the incorporation of specific carotenoids into micelles, could ultimately be considered as a strategy for the development of functional foods. In addition, the study is focused on a matrix-free material, in which incorporation into micelles and the respective bioaccessibility are not affected by the food matrix, as has been evaluated in other studies. The goal of this study was to isolate different carrot root chromoplasts fractions by means of sucrose gradients to assess whether such compositional and structural differences are accompanied by marked differences in the profile of carotenoids and their bioaccessibility.

## 2. Results and Discussion

### 2.1. Optical Microscopy

**Figure 1:**
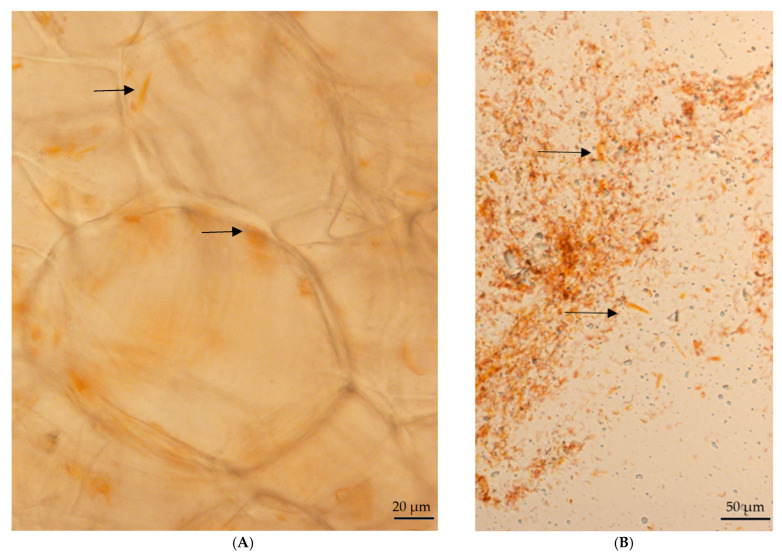
Light micrographs of (**A**) free-hand sections of carrot root crystalloid chromoplast fresh carrot and (**B**) isolated chromoplast fraction before sucrose density gradient centrifugation. Arrows mark chromoplasts.

Photographs taken with a light microscope are shown in Figure 1, in which the ultrastructure of fresh carrot (A) and isolated chromoplast fraction (B) can be seen. Large needle-shaped crystalloid chromoplasts of β-carotene-rich carrot root can be readily observed, as previously reported by other authors [1,14,15]. In fresh tissue, chromoplasts appear embedded within the cellular matrix, maintaining their structural integrity. In contrast, in the isolated fraction, chromoplasts are more dispersed, likely due to the loss of surrounding cellular components and potential alterations in membrane organization during the isolation process. These differences highlight the structural heterogeneity of chromoplasts and their dynamic nature within the cellular environment.

Jeffery et al. (2012) [11] described the carrot cell wall as a very fibrous, compact and stratified cell wall, with a thickness of 424–954 nm and cell sizes between 42 and 6274 µm^2^. They also described the presence of chromoplasts filled with an electron dense material and a layer of pectin that could reduce the porosity of the cell wall. 

The relative size of the chromoplasts in the different zones of the sucrose gradient would be approximately as follows: 60%: 200–300 nm, 50%: 300–400 nm, 40%: 400–500 nm, 30%: 450–500 nm and 15%: 500 nm.

The most prevalent chromoplast type in carrot roots is the crystalloid chromoplast, which contains solid-crystalline carotenoids. These structures are primarily composed of β-carotene, which constitutes about 80% of total carotenoids although its proportion may vary depending on the carrot variety and other factors [16]. It is reported that crystalloid chromoplasts in carrot root possess different shapes. With an increase in carotenoid concentration, they tend to crystallize inside the chromoplast, where large crystals may distort the shape and size of the chromoplast [16]. Interestingly, studies on carrot callus cultures have revealed the presence of all four main types of chromoplasts: crystalloid, globular, tubular, and membranous. This unusual diversity indicates a complex plastid biogenesis in carrot tissue [16]. 

### 2.2. Isolation of Carrot Chromoplasts

The separation of chromoplasts by sucrose gradient centrifugation is based on differences in density and physical properties resulting from their molecular and structural composition. During high-speed centrifugation, chromoplasts migrate to the position in the gradient where their density matches that of the medium. In particular, the flotation technique in a sucrose gradient allows chromoplasts, being less dense than other organelles like mitochondria, to float toward regions of lower density. This facilitates the attainment of highly pure chromoplast fractions, as denser contaminants remain at lower levels of the gradient. This methodology is essential for precise biochemical studies, as it overcomes the limitations of conventional centrifugation, where effective separation and contaminant elimination are less successful because organelles migrate from top to bottom in the gradient. Therefore, flotation centrifugation in a sucrose gradient is an indispensable requirement for isolating pure chromoplast fractions and accurately analysing their composition and function [17].

**Figure 2:**
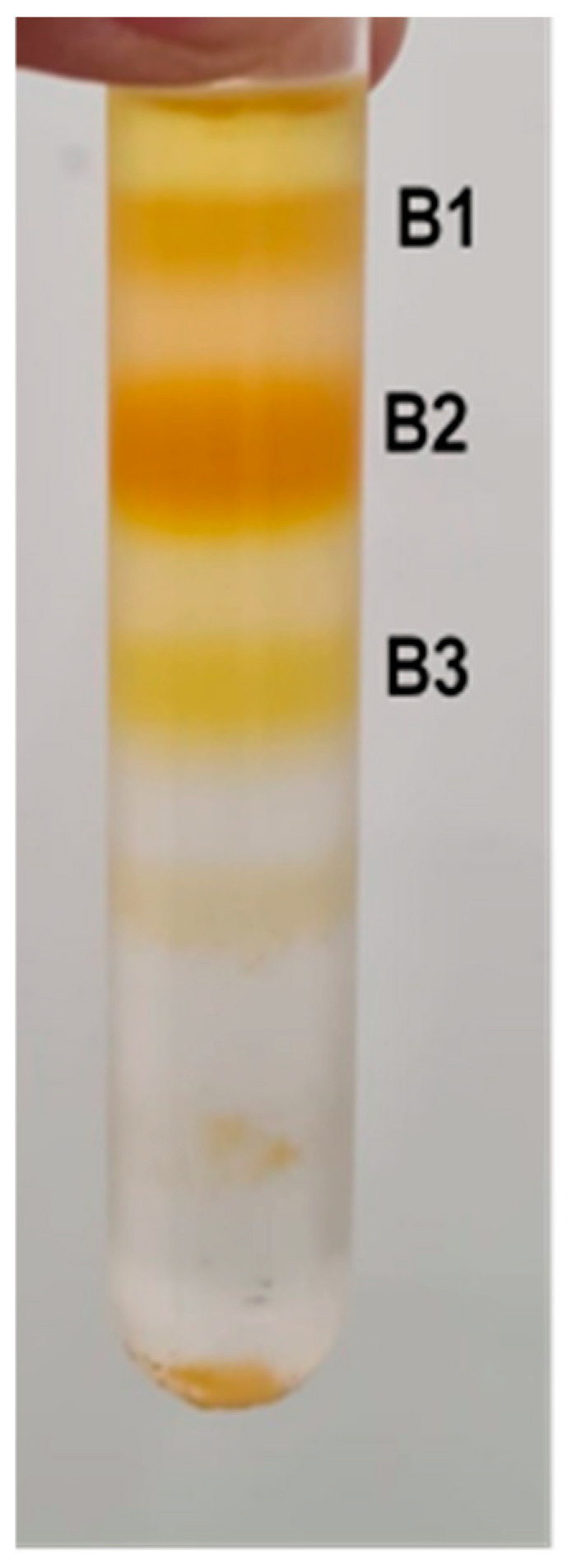
Chromoplast bands from carrot root obtained by density gradient centrifugation. Three distinct bands were obtained corresponding to the sucrose gradient zone of 15%/30%, (B1), 30%/40% (B2) and 40%/50% (B3) interfaces.

Three distinct bands were obtained (Figure 2) corresponding to the sucrose gradient zone of 15%/30%,(B1), 30%/40% (B2) and 40%/50% (B3) interfaces. The bands were carefully separated and further processed for carotenoid analysis and bioaccessibility assessment.

The separation of chromoplast fragments into three distinct bands in the sucrose gradient is likely influenced by differences in their lipid-protein composition and structural organization. Chromoplasts are heterogeneous organelles containing specialized subdomains, such as plastoglobules, lipid bodies, and remnants of thylakoids, which may contribute to variations in density. Additionally, differences in carotenoid aggregation state, such as crystalline versus dissolved forms, may also affect membrane packing and buoyancy. Further studies using lipidomics, proteomics, or spectroscopic techniques could provide deeper insights into the biochemical factors underlying this separation.

### 2.3. Carotenoid Content

First, the fresh carrot sample from which the chromoplasts were isolated was analysed. Table 1 shows the concentration, bioaccessible content and bioaccessibility of the carotenoids in this sample. The chromatographic profile is shown in Figure S1.

The concentration of total carotenoids in fresh carrot was 131.05 µg/g carrot, of which 119.13 µg corresponded to the coloured carotenoids (ζ-carotene, lutein, α-carotene and β-carotene) and the rest to colourless phytoene and phytofluene. However, after digestion, the bioaccessible content of the coloured carotenoids was 2.726 µg, while that of the colourless carotenoids was 6.06 µg. This means that although the coloured carotenoids (especially α-carotene and β-carotene) are the major carotenoids in carrot, their bioaccessibility was only 2.3% compared to 50.8% for the colourless carotenoids. This behaviour is in line with that reported by Benítez González et al. (2024) [18], who reported the low bioaccessibility of the major carotenoids in carrot compared to the colourless ones and compared different cooking methods to improve the bioaccessibility of these carotenoids.

Regarding the different chromoplast fractions isolated, the distribution of carotenoids in the bands was similar. The colourless carotenoids phytoene and phytofluene were identified in all bands, as well as ζ-carotene, lutein, and the provitamins A α-carotene and β-carotene. 

The concentrations and percentages of carotenoids found in each isolated band (before digestion) are summarized in Table 2. In the isolated bands, no significant differences in carotenoid concentration were found, although B1 was the fraction with the lowest concentration of coloured and total carotenoids. The exception to this trend was lutein, where there was significant difference in concentration between the bands, with the lowest concentration found in B3. 

It is interesting to note that the percentages remain relatively constant in the isolated bands. The colourless carotenoids phytoene and phytofluene accounted for approximately 12% of the total carotenoids and ζ-carotene for approximately 3%. The macular carotenoid lutein is prominent and is present in all bands, but its concentration decreases as the sucrose gradient increases, being 1.61% in B1 and only 0.77% in B3. As expected, the provitamin A carotenoids (α-carotene and β-carotene) were the most abundant in all bands, together accounting for almost 85% of the total carotenoids in each band. Although the bands correspond to chromoplasts of different densities, the results obtained seem to suggest the possible existence of some internal cellular mechanisms that regulate the synthesis or accumulation of carotenoids in the chromoplasts of the isolated fractions. In this sense, chromoplasts could synthesise or accumulate carotenoids in the same relative proportions, regardless of their density or structure.

The results suggest that B1 contains the lowest concentration of carotenoids, indicating that the less dense chromoplasts floating in the lower sucrose density contain fewer carotenoids. The higher density of bands B2 and B3 suggests that they represent chromoplasts with a higher degree of development or maturity and that they are able to accumulate a higher concentration of carotenoids [19,20].

It is possible that some plastoglobuli can be located in the upper layers, being less dense structures compared to crystalline structures or thylakoid-associated ones [21]. A higher number of globular structures containing lutein might be found in the upper band, corresponding to the fraction with the lowest density. The band with higher contents of carotenoids may also contain larger crystals due to the crystallization of β- and α-carotene. Additionally, the association with proteins in the different chromoplasts may affect the physical properties that define their location in the various bands [6].

While crystalloid chromoplasts dominate in carrot roots, other types are also present. Globular chromoplasts, containing lipid-dissolved carotenoids in plastoglobuli, have been observed in carrot root cells in smaller numbers and sizes [16]. These structures may be involved in the accumulation of soluble carotenoids and other lipophilic compounds. A relationship between chromoplast type and carotenoid content and profiles has been previously described [22]. It is usual that one type of chromoplast dominates, but they may coexist with other types, even in the same cell. It is considered that the occurrence of different chromoplast types may be result of differentiation processes or to the transition between plastid types [23]. Different chromoplast types were observed in carrot callus tissues, although crystalloid chromoplasts were the predominant. Also, globular chromoplasts, containing plastoglobuli and membrane fragments, were observed in small numbers in carrot root tissue [16]. Plastoglobuli are considered primary carotenoid-containing structures in chromoplasts and serve as lipid storage [24].

### 2.4. Bioaccessibility and Carotenoid Bioaccesible Content

Table 3 summarizes the concentration and proportion of micellar carotenoids in each band.

Contrary to the bands analysed before digestion, colourless carotenoids were found in the highest proportion, with an average of 41.7% phytoene and 28.4% phytofluene, representing almost 70% of all carotenoids. Moreover, in this case, the percentage of carotenoids present in each of the fractions is constant, as observed in the bands before digestion.

The provitamin A carotenoids and ζ-carotene were similarly distributed in the three bands, with the concentration of ζ-carotene being approximately 19%, α-carotene almost 8% and β-carotene only 0.2%. LUT, on the other hand, showed an uneven distribution ranging from 3.2% in the B1 band, 4.1% in the B2 band and 0.5% in the B3 band.

In the samples before digestion, the concentration of the provitamin carotenoids α-carotene and β-carotene was higher than that of the colourless carotenoids phytoene and phytofluene in the isolated bands. α-carotene was between 8.4 and 12.9 times higher than phytoene and between 5.1 and 10.8 times higher than phytofluene; in the case of β-carotene, this ratio was between 8.4 and 14.6 times that of phytoene and 4.4 to 10.9 times that of phytofluene. This trend changed in the case of the samples after digestion, where the CBC of the colourless carotenoids was higher than that of the provitamins, with phytoene between 3.8–5.5 times and phytofluene between 1.6–4.0 times higher than α-carotene. What is particularly striking is what happens with β-carotene, whose low bioavailability is well known; this, combined with the high bioavailability of phytoene, means that in the isolated bands, the CBC of phytoene is, on average, 184 times higher than that of β-carotene, and that of phytofluene is, on average, 126 times higher. 

The results suggest that carotenoids with simpler and fewer conjugated double bonds (such as phytoene and phytofluene) have significantly higher bioaccessibility than the more complex and cyclic ones (α-carotene and β-carotene), probably because they have a lower tendency to crystallize, higher solubility in digestive fluids and easier micellarization. Furthermore, the colourless carotenoids tend to be more abundant in cis-isomers, which are more bioaccessible than their all-trans counterparts [25,26]. On the other hand, the differences observed between α-carotene and β-carotene (structural isomers) could be due to the fact that α-carotene has a β- and α- ring in its structure, whereas β-carotene has two β-rings. These structural differences may also favour the crystallisation of β-carotene in chromoplasts.

This trend is consistent with that reported by Benítez-González et al. (2024) [27], who reported that in Tenebrio molitor powder fed with carrot as a source of moisture and carotenoids, the highest CBCs were reported for colourless carotenoids. In addition, Benítez-González et al. (2024) [18] also analysed the content of bioaccessible carotenoids in carrots cooked by different methods and showed that the micellar content of colourless carotenoids in raw carrots was higher than 80%. 

The micellar carotenoid content was also directly proportional to the sucrose gradient, with the B1 band having the lowest carotenoid concentration and the B3 band having the highest. There were no statistically significant differences between the B1 and B2 bands (*p* > 0.05), while there were significant differences between the B1, B2 and B3 bands (*p* < 0.05) for all carotenoids.

The highest concentrations of total carotenoids were found in B3, with a concentration 2.8 and 2.5 times higher than in B1 and B2, respectively. This ratio was even higher in total colourless carotenoids, where the concentration of B3 was 3.0 and 2.7 times higher than in B1 and B2, respectively.

**Figure 3:**
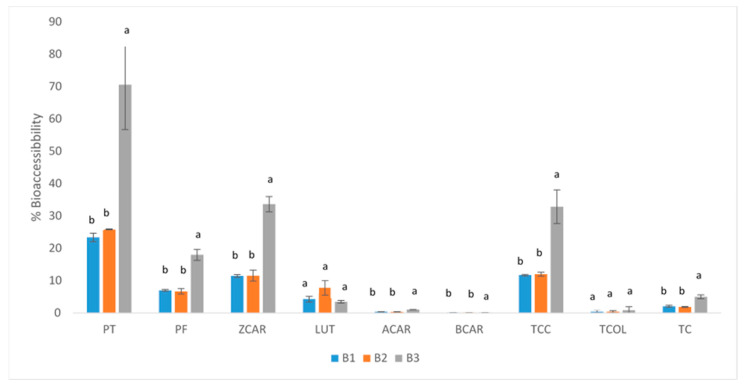
Carotenoid bioaccessibility (%) values. Different letters indicate significant differences (*p* < 0.05) in the corresponding compounds among the different bands. PT: phytoene; PF: phytofluene; TCC: total colourless (Sum of PT and PF); ZCAR: ζ-carotene; LUT: lutein; ACAR: α-carotene; BCAR: β-carotene; TCOL: total coloured carotenoids (ZCAR + LUT + ACAR + BCAR); TC: total carotenoids (TCC + TCOL).

Regarding the percentage of carotenoid bioaccessibility (Figure 3) in the different bands, it was observed that the percentages followed a descending order: phytoene > ζ-carotene > phytofluene > lutein > α-carotene > β-carotene. Overall, the percentage of bioaccessibility was significantly higher in the 40% sucrose band in all cases, except for lutein, which showed irregular behaviour.

Micellarization extent is thought to be inversely proportional to the hydrophobicity of carotenoids, where carotenes are less polar and arrange in in the core of lipid droplets, while xanthophylls, which are more polar, are deposited in the outer layer together with proteins, phospholipids, and fatty acids. This could explain that lutein has been found to be more easily incorporated into micelles in comparison to β-carotene [28,29,30]. However, phytoene, followed by phytofluene, were reported to be the carotenoids with the highest bioaccessibility in juices of tomato, carrot, blood orange and apricot, despite the fact that they are carotenes. This suggests that, apart from the hydrophobicity of carotenoids, the number and arrangement of conjugated double bonds, which affects the conformation of the molecules, are also important factors in relation to the bioaccessibility of carotenoids [31]. Also, the crystalloid chromoplasts in carrots typically form needle-, ribbon-, or tube-like crystals, which result from the accumulation of β-carotene and maybe α-carotene have an important impact in bioaccessibility. These crystalline structures reduce the bioaccessibility of the carotenoids present in them, as they are less soluble and more difficult to release [6].

All these trends coincide with those reported in previous works [32], showing that although provitamin A carotenoids are the most abundant in carrots, only a very small amount (0.5%) is potentially absorbed by the organism. However, colourless carotenoids, whose potential health-promoting biological actions have recently been eliciting increased interest lately [33,34], account for almost 80% of the total bioavailable carotenoids present in carrots, despite their lower concentration [18].

Schweiggert et al. (2012) [1] studied the influence of chromoplast morphology on the bioaccessibility of carotenoids from different matrices. They found that, in descending order, the bioaccessibility of β-carotene was highest in mango, with 10.1%, while papaya, tomato and carrot had only 0.5%. In mango, carotenoids are deposited in liquid crystalline tubular elements dissolved in lipids of the mesocarp chromoplasts, suggesting that the release and micellarisation of carotenoids from these structures was greater than those from large solid crystalline substructures, as observed in carrot. In addition, the authors proposed that the presence of lycopene crystallites in papaya may protect the chromoplast during digestion, which would explain the greater bioaccessibility of β-carotene in mango.

Furthermore, Schweiggert et al. (2017) [10], in their comprehensive review on the deposition form of carotenoids and its impact on bioaccessibility, stated that in carrots (mainly rich in α-carotene and β-carotene), carotenoids are deposited in crystalloid chromoplasts with large crystals, often up to 15 mm in length [35], and this form of crystalline deposition is associated with their poor bioavailability. Therefore, to improve the bioavailability of these compounds, it is recommended to add some form of fat to the diet, as this addition leads to the dissolution of crystalline carotenoids, which is a prerequisite for their incorporation into mixed micelles and thus promotes more efficient absorption into the enterocyte [32,36]. In addition to fat incorporation, different culinary processes improve the bioaccessibility of carrot carotenoids by up to 40-fold [18,37].

## 3. Materials and Methods

### 3.1. Samples

Carrots (*Daucus carota* cv ‘*Nantesa*’) were purchased from a local market (Ecofrutas, Seville, Spain).

### 3.2. Chemicals

All reagents required for the preparation of buffers A and B were of the highest purity for each component and were purchased from Merck (Merck, Darmstadt, Germany) and Panreac (Barcelona, Spain). Enzymes and all reagents necessary for the preparation of the solutions used in the bioaccessibility test (saliva, gastric and intestinal phases) were purchased from Sigma-Aldrich (St. Louis, MO, USA). The mobile phase used for chromatographic analysis consisted of methanol (MeOH), methyl tert-butyl ether (MTBE), and ethyl acetate, all of HPLC grade (supplied by Merck), and ultrapure water (NANOpure Diamond™ system, Barnstead Inc., Dubuque, IA, USA). Standards for carotenoids were purchased from Sigma-Aldrich (purity > 95%), while phytofluene was purchased from CaroteNature GmbH (Münsingen, Switzerland).

### 3.3. Isolation of Carrot Chromoplasts

Chromoplasts were isolated according to the method described by Angaman et al. (2012) [13]. Fresh carrots (30–40 g) were peeled and chopped into small pieces in a beaker, to which two volumes of buffer A (100 mM tris-HCl pH 8.2, 0.33M sorbitol, 2 mM MgCl_2_, 10 mM KCl, 8 mM EDTA, 10 mM ascorbic acid, 5 mM L-cysteine, 1 mM PMSF, 1% PVPP, 1 mM DTT) were added. The mixture was homogenized using a domestic blender under three short pulses at low speed. The homogenate was filtered through a funnel and an 8-layer cheesecloth. The resulting filtrate was then filtered again through a 2-layer cheesecloth. The biomass retained on the cheesecloth was subjected to the same procedure. The collected filtrates were pooled and centrifuged at 4 °C for 2 min at 200× *g*, and the supernatant was collected. This supernatant was centrifuged again, this time at 5000× *g* for 10 min. After centrifugation, the supernatant was discarded and the resulting pellet was resuspended in 50 mL of buffer B (same composition as buffer A but without addition of PVPP). This suspension was centrifuged again at 5000× *g* for 10 min, after which the supernatant was discarded. The final pellet was resuspended in 2 mL of buffer B. A 13 mL ultracentrifuge tube was prepared with 2 mL of each sucrose gradient concentration, arranged from highest to lowest (60%, 50%, 40%, 30% and 15%). When all layers were added, 1 mL of sample was added to the tube and the gradient was centrifuged in a Beckman ultracentrifuge at 4 °C for 1 h at 100,000× *g* using a swing-out bucket rotor (SW 28 rotor).

After centrifugation, the tubes were carefully removed without disturbing the gradient. Using a glass Pasteur pipette, each coloured band was extracted and transferred to separate Eppendorf tubes. All fractions were washed with an equal volume of buffer B and centrifuged at 5000× *g* for 10 min. The supernatant was then discarded, leaving the chromoplasts in the pellet of each tube.

### 3.4. Light Microscopy

Microscopic images were taken at the Microscopy Service of the University of Seville. An Olympus BX61 epifluorescence microscope (Olympus Corporation, Tokyo, Japan) was used for light microscopy. The fresh carrot sample was cut freehand, while the combination of the isolated chromoplast fraction was dissolved in a small amount of distilled water. Both samples were mounted on a glass slide with a standard microscope coverslip and observed under the microscope without staining.

### 3.5. Simulated In Vitro Digestion Method

To assess the bioaccessibility of carotenoids from the isolated bands, an in vitro static gastrointestinal model simulating digestion, following the INFOGEST 2.0 protocol by Brodkorb et al., (2019), was used [38]; although the in vitro digestion study has limitations, such as the absence of microbiota, it is a widely used and reliable method for assessing the bioaccessibility of carotenoids. 

All parameters, including the composition of the simulated fluids, enzymes, dilutions, incubation time, pH and temperature, as well as the use of N_2_ (to prevent oxidation), were consistent with those outlined in this protocol. To simulate oral digestion, samples were mixed with salivary fluid containing α-amylase at an adjusted concentration and the pH was maintained at 7.0 by the addition of HCl or NaOH. The mixture was incubated at 37 °C for two minutes with constant agitation to mimic the mechanical conditions of chewing. For gastric digestion, the material was then mixed with simulated gastric fluid containing pepsin (2000 U/mL) and the pH was adjusted to 3.0 by the addition of HCl. This mixture was incubated for two hours at 37 °C with constant agitation to simulate peristaltic movements. Intestinal digestion was simulated by adding simulated intestinal fluid containing pancreatic enzymes (100 U/mL lipase) and bile salts (10 mmol/L) to the gastric contents and adjusting the pH to 7.0 with NaOH. Samples were incubated at 37 °C for two hours with continuous agitation to simulate small intestinal conditions.

At the end of the digestion process, the micelle-containing fraction was isolated by centrifugation of the samples at 3900× *g* for 20 min at 4 °C using an Allegra X-12R centrifuge (Beckman Coulter, Brea, CA, USA). The supernatants were filtered through a 0.22 µm nylon membrane (Agilent Technologies, Santa Clara, CA, USA) and the recovered micellar fractions were stored at −20 °C in a nitrogen atmosphere until analysis. All samples were run in quadruplicate.

### 3.6. Carotenoid Content

Carotenoid extraction from the samples was performed according to the protocol validated by Stinco et al. (2019) [39]. For undigested samples, 1 mL diethyl ether was added to each sample (on triplicate) followed by agitation using an automatic vortex shaker at 2500 rpm for 5 min. The samples were then centrifuged at 3900× *g* and 4 °C for 5 min. The coloured fraction was collected, and the residue was subjected to the same procedure until no colour remained. For the digested samples, the micellar fraction (10–12 mL) was homogenised with 5 mL diethyl ether using an UltraTurrax T25 homogeniser at 24,000 rpm for 5 min. The mixture was then centrifuged at 3900× *g* for 5 min at 4 °C. The supernatant was collected, and the remaining residue was extracted again using the same protocol until no colour remained.

The collected fractions were concentrated to dryness using a vacuum rotary concentrator (Eppendorf Concentrator Plus, Hamburg, Germany).

Before HPLC analyses, dry extracts were redissolved in ethyl acetate, centrifuged at 18,000 × *g* for 5 min at 4 °C and transferred to amber glass vials. Carotenoids were determined on an Agilent 1260 chromatograph (Agilent Technologies, Palo Alto, CA, USA) equipped with a diode array detector (DAD) and a C30 YMC column (3 μm, 250 × 4.6 mm) (YMC, Wilmington, NC, USA) at 20 °C. Chromatographic conditions (e.g., linear gradient, flow rate, DAD conditions) were established according to the method validated by Stinco et al. (2019) [39]. The quantification was carried out by external calibration from the areas of the chromatographic peaks obtained by DAD detection at the following wavelengths: 285 nm for phytoene, 350 nm for phytofluene, 410 nm for ζ-carotene and 450 nm for the rest of the CARS (lutein, α-carotene and β-carotene).

### 3.7. Bioaccessibility and Carotenoid Bioaccessible Content

The bioaccessibility for each compound is the ratio of the carotenoid content incorporated into the micelles to the carotenoid content of the sample before digestion, in percentage (%BIO), and was calculated according to Equation (1):

(1) $$\%BIO=\frac{Carotenoids\;Micellar\;Fraction}{Carotenoids\;Undigested\;fraction}\times 100$$

The bioaccessible carotenoid content (CBC) is the amount of carotenoids incorporated into micelles and available for absorption. In other words, it provides an estimate of the amount of carotenoids that can potentially be absorbed and made bioavailable from a given portion of food. This information is more meaningful than bioaccessibility (usually expressed as a percentage) because CBC provides a more practical insight into potential bioavailability. As the food was not directly analysed in this study, we expressed this amount in µg of carotenoid/g, referring to the amount of band isolated in each percentage of the sucrose gradient.

### 3.8. Statistical Analysis

Results were expressed as mean and standard deviation of three independent determinations. One-way analysis of variance (ANOVA) was used to compare the means. Statistical analysis was performed with Infostat 2020e software. Differences were considered significant at *(p* < 0.05) using Tukey’s multiple comparison procedure.

## 4. Conclusions

The present work advances the state of the art by isolating and analysing the bioaccessibility and CBC (including colourless carotenoids) in the different chromoplast fractions rather than the bioaccessibility of the matrix from which they originate. The results showed that the proportion of carotenoid content in each isolated chromoplast band was constant, suggesting that chromoplasts internally regulate the synthesis or accumulation of carotenoids, maintaining relative proportions independent of their density or structure.

This study provides further evidence that the structural characteristics of carrot carotenoids—in particular, the number of conjugated double bonds, the presence of terminal cyclic groups and the tendency to aggregate to form crystals—play a crucial role in their bioaccessibility, in this case, from different chromoplast fractions. The results show that linear carotenoids with few conjugated double bonds, such as phytoene and phytofluene (three and five conjugated double bonds, respectively) have significantly higher bioaccessibility from chromoplasts than more complex, cyclic carotenoids such as β-carotene. This study adds further evidence that chromoplasts are key structures influencing the greater or lesser bioavailability of carotenoids and underscores that the bioaccessibility and bioaccessible content of the traditionally overlooked colourless carotenoids phytoene and phytofluene from carrot chromoplasts is remarkable and ostensibly higher relative to those of the major carrot carotenoids α- and β-carotene. Specifically, the bioaccessible content of the colourless carotenoids in the isolated chromoplast bands was almost 100 times higher than that of the provitamin A carotenoids. Further research to gain insight into the localization and organization of these carotenoids within plant chromoplasts is warranted.

## Figures and Tables

**Table 1:** Contents of carotenoids (CC) (µg carotenoid/g fresh carrot), carotenoid bioaccesible content (CBC) (µg carotenoid/g fresh carrot) and bioaccessibility (% BIO).

Carotenoid	CC	CBC	% BIO
phytoene	6.723 ± 0.005	4.276 ± 0.431	63.603 ± 9.669
phytofluene	5.202 ± 0.284	1.782 ± 0.081	34.256 ± 6.872
TCC	11.925 ± 0.279	6.058 ± 0.512	50.769 ± 3.107
ζ-carotene	4.804 ± 0.200	0.499 ± 0.013	10.387 ± 1.322
lutein	1.372 ± 0.114	0.312 ± 0.014	22.740 ± 5.120
α-carotene	56.241 ± 1.279	1.134 ± 0.127	2.016 ± 0.244
β-carotene	56.710 ± 2.792	0.781 ± 0.103	1.378 ± 0.193
TCOL	119.127 ± 3.757	2.726 ± 0.258	2.293 ± 0.289
TC	131.052 ± 3.478	8.785 ± 0.770	6.703 ± 0.766

Data represented as mean values ± standard deviation (*n* = 3) in µg carotenoid/g fresh carrot. TCC: Total Colourless (Sum of phytoene and phytofluene); TCOL: total coloured carotenoids (ζ-carotene+ lutein+ α-carotene + β-carotene), TC: total carotenoids (TCC + TCOL).

**Table 2:** Contents of carotenoids (µg carotenoid/isolated band) and their respective percentages in the bands corresponding to different fractions of sucrose gradients of carrot root isolated chromoplasts *.

Carotenoid	Band 1 (B1)	Band 2 (B2)	Band 3 (B3)
phytoene	1.509 ± 0.056 (3.48%) ^a^	1.589 ± 0.180 (2.88%) ^a^	1.574 ± 0.024 (3.08%) ^a^
phytofluene	3.782 ± 0.066 (8.71%) ^a^	4.135 ± 0.422 (7.49%) ^a^	3.993 ± 0.048 (7.81%) ^a^
TCC	5.291 ± 0.122 (12.19%) ^a^	5.725 ± 0.602 (10.37%) ^a^	5.567 ± 0.072 (10.89%) ^a^
ζ-carotene	1.533 ± 0.043 (3.53%) ^a^	1.706 ± 0.201 (3.10%) ^a^	1.458 ± 0.011 (2.85%) ^a^
lutein	0.740 ± 0.079 (1.70%) ^a^	0.539 ± 0.107 (0.97%) ^ab^	0.392 ± 0.006 (0.77%) ^b^
α-carotene	19.374 ± 3.165 (44.62%) ^a^	25.430 ± 3.800 (46.06%) ^a^	20.720 ± 0.465 (40.52%) ^a^
β-carotene	16.478 ± 2.524 (37.95%) ^a^	21.812 ± 3.074 (39.51%) ^a^	22.993 ± 0.528 (44.97%) ^a^
TCOL	38.125 ± 5.652 (87.81%) ^a^	49.487 ± 7.183 (89.63%) ^a^	45.563 ± 1.010 (89.11%) ^a^
TC	43.416 ± 5.531 ^a^	55.211 ± 7.785 ^a^	51.130 ± 1.082 ^a^

* Data represented as mean values ± standard deviation (*n* = 3) in µg carotenoid/isolated band carotenoids in the fraction. Different letters indicate significant differences (*p* < 0.05) of the means for each line. TCC: total colourless (sum of phytoene and phytofluene); TCOL: total coloured carotenoids (ζ-carotene+ lutein+ α-carotene + β-carotene); TC: total carotenoids (TCC + TCOL).

**Table 3:** Concentration (µg carotenoid/isolated band) and proportion of micellar carotenoids in each isolated band (%).

Carotenoid	Band 1 (B1)	Band 2 (B2)	Band 3 (B3)
phytoene	0.353 ± 0.033 (40.47%) ^a^	0.410 ± 0.044 (41.10%) ^a^	1.113 ± 0.236 (43.38%) ^b^
phytofluene	0.264 ± 0.005 (29.45%) ^a^	0.273 ± 0.007 (27.47%) ^a^	0.717 ± 0.076 (28.16%) ^b^
TCC	0.617 ± 0.028 (69.19%) ^a^	0.683 ± 0.038 (68.57%)^a^	1.830 ± 0.312 (71.54%) ^b^
ζ-carotene	0.175 ± 0.011 (19.08%) ^a^	0.195 ± 0.006 (19.58%) ^a^	0.491 ± 0.038 (19.30%) ^b^
lutein	0.031 ± 0.003 (3.23%) ^a^	0.041 ± 0.004(4.10%) ^a^	0.013 ± 0.001 (0.54%) ^b^
α-carotene	0.066 ± 0.002 (7.53%) ^a^	0.075 ± 0.001 (7.53%) ^a^	0.212 ± 0.004 (8.37%) ^b^
β-carotene	0.002 ± 0.001 (0.24%) ^a^	0.002 ± 0.001 (0.21%) ^a^	0.006 ± 0.001 (0.25%) ^b^
TCOL	0.275 ± 0.017 (30.81%)^a^	0.313 ± 0.010 (31.43%) ^a^	0.722 ± 0.032 (28.46%) ^b^
TC	0.892 ± 0.011 ^a^	0.996 ± 0.028 ^a^	2.552 ± 0.345 ^b^

Data represented as mean values ± standard deviation (*n* = 4) in µg carotenoid/isolated band in the fraction incorporated into micelles. Different letters indicate significant differences (*p* < 0.05) of the means for each line. TCC: Total Colourless (Sum of phytoene and phytofluene); TCOL: total coloured carotenoids (ζ-carotene+ lutein+ α-carotene + β-carotene) TC: total carotenoids (TCC + TCOL).

## Data Availability

The data supporting this study are available upon request.

## Supplementary Materials

The following supporting information can be downloaded at: https://www.mdpi.com/article/10.3390/molecules30061267/s1, Figure S1: Chromatographic profile of carrot roots.

## Abbreviations

The following abbreviations are used in this manuscript:

%BIO	Bioaccessibility percentage
CBC	Carotenoid Bioaccessible Content
BCAR	β-carotene
ACAR	α-carotene
ZCAR	ζ-carotene
LUT	Lutein
PT	Phytoene
PF	Phytofluene
TCC	Total Colourless Carotenoids
TCOL	Total Coloured Carotenoids
TC	Total Carotenoids

## References

[1] Schweiggert R.M., Mezger D., Schimpf F., Steingass C.B., Carle R. (2012). Influence of Chromoplast Morphology on Carotenoid Bioaccessibility of Carrot, Mango, Papaya, and Tomato. Food Chem..

[2] Meléndez-Martínez A.J., Stinco C.M., Mapelli-Brahm P. (2019). Skin Carotenoids in Public Health and Nutricosmetics: The Emerging Roles and Applications of the UV Radiation-Absorbing Colourless Carotenoids Phytoene and Phytofluene. Nutrients.

[3] Killeit U. (2021). European Database of Carotenoid Levels in Food. Dtsch. Leb..

[4] Song H., Lu Q., Song T., Gao C., Zhu W., Guo X. (2024). Study on the Mechanism of Carotenoid Production and Accumulation in Orange Red Carrot (*Daucus carota* L.). Sci. Hortic..

[5] Chevalier W., Moussa S.A., Ottoni M.M.N., Dubois-Laurent C., Huet S., Aubert C., Desnoues E., Navez B., Cottet V., Chalot G. (2022). Evaluation of Pedoclimatic Factors and Cultural Practices Effects on Carotenoid and Sugar Content in Carrot Root. Eur. J. Agron..

[6] Wang Y.Q., Yang Y., Fei Z., Yuan H., Fish T., Thannhauser T.W., Mazourek M., Kochian L.V., Wang X., Li L. (2013). Proteomic Analysis of Chromoplasts from Six Crop Species Reveals Insights into Chromoplast Function and Development. J. Exp. Bot..

[7] Esquivel P., Schweiggert R.M., Chacón-Ordóñez T., Steingass C.B., Carle R., Jiménez V.M. (2019). Carotenoid Assembly in Fruits and Vegetables. Food Chem. Funct. Anal..

[8] Egea I., Barsan C., Bian W., Purgatto E., Latché A., Chervin C., Bouzayen M., Pech J.C. (2010). Chromoplast Differentiation: Current Status and Perspectives. Plant Cell Physiol..

[9] Carrillo C., Buvé C., Panozzo A., Grauwet T., Hendrickx M. (2017). Role of Structural Barriers in the in Vitro Bioaccessibility of Anthocyanins in Comparison with Carotenoids. Food Chem..

[10] Schweiggert R.M., Carle R. (2017). Carotenoid Deposition in Plant and Animal Foods and Its Impact on Bioavailability. Crit. Rev. Food Sci. Nutr..

[11] Jeffery J., Holzenburg A., King S. (2012). Physical Barriers to Carotenoid Bioaccessibility. Ultrastructure Survey of Chromoplast and Cell Wall Morphology in Nine Carotenoid-Containing Fruits and Vegetables. J. Sci. Food Agric..

[12] Li L., Yuan H. (2013). Chromoplast Biogenesis and Carotenoid Accumulation. Arch. Biochem. Biophys..

[13] Angaman D.M., Petrizzo R., Hernández-Gras F., Romero-Segura C., Pateraki I., Busquets M., Boronat A. (2012). Precursor Uptake Assays and Metabolic Analyses in Isolated Tomato Fruit Chromoplasts. Plant Methods.

[14] Berry H.M., Nogueira M., Drapal M., Almeida J., Perez-Fons L., Enfissi E.M.A., Fraser P.D. (2022). Isolation and Characterization of Sub-Plastidial Fractions from Carotenoid Rich Fruits.

[15] Vásquez-Caicedo A.L., Heller A., Neidhart S., Carle R. (2006). Chromoplast Morphology and β-Carotene Accumulation during Postharvest Ripening of Mango Cv. “Tommy Atkins”. J. Agric. Food Chem..

[16] Kim J.E., Rensing K.H., Douglas C.J., Cheng K.M. (2010). Chromoplasts Ultrastructure and Estimated Carotene Content in Root Secondary Phloem of Different Carrot Varieties. Planta.

[17] Liedvogel B. (1987). Isolation of Membranous Chromoplast from Daffodil Flowers. Methods Enzymol..

[18] Benítez-González A.M., Stinco C.M., Rodríguez-Pulido F.J., Vicario I.M., Meléndez-Martínez A.J. (2024). Towards More Sustainable Cooking Practices to Increase the Bioaccessibility of Colourless and Provitamin A Carotenoids in Cooked Carrots. Food Funct..

[19] Lu S., Li L. (2008). Carotenoid Metabolism: Biosynthesis, Regulation, and Beyond. J. Integr. Plant Biol..

[20] Li L., Yuan H., Zeng Y., Xu Q. (2016). Plastids and Carotenoid Accumulation. Carotenoids in Nature. Subcellular Biochemistry.

[21] Coulon D., Bréhélin C. (2021). Isolation of Plastoglobules for Lipid Analyses. Methods Mol. Biol..

[22] Oleszkiewicz T., Klimek-Chodacka M., Milewska-Hendel A., Zubko M., Stróż D., Kurczyńska E., Boba A., Szopa J., Baranski R. (2018). Unique Chromoplast Organisation and Carotenoid Gene Expression in Carotenoid-Rich Carrot Callus. Planta.

[23] Pyke K. (2007). Plastid Biogenesis and Differentiation. Top. Curr. Genet..

[24] Lundquist P.K. (2023). Chromoplast Differentiation: A Central Role for Plastoglobule Lipid Droplets Comes into Focus. New Phytol..

[25] Mapelli-Brahm P., Corte-Real J., Meléndez-Martínez A.J., Bohn T. (2017). Bioaccessibility of Phytoene and Phytofluene Is Superior to Other Carotenoids from Selected Fruit and Vegetable Juices. Food Chem..

[26] Meléndez-Martínez A.J., Paulino M., Stinco C.M., Mapelli-Brahm P., Wang X.-D. (2014). Study of the Time-Course of Cis/Trans (Z/E) Isomerization of Lycopene, Phytoene, and Phytofluene from Tomato. J. Agric. Food Chem..

[27] Benítez-González A.M., Aguilera-Velázquez J.R., Bautista Palomas J., Meléndez-Martínez A.J. (2024). Evaluation of Carrot and Agroindustrial Residues for Obtaining Tenebrio Molitor (Yellow Mealworm) Powder Enriched in Bioaccessible Provitamin A and Colourless Carotenoids. LWT.

[28] Mashurabad P.C., Palika R., Jyrwa Y.W., Bhaskarachary K., Pullakhandam R. (2017). Dietary Fat Composition, Food Matrix and Relative Polarity Modulate the Micellarization and Intestinal Uptake of Carotenoids from Vegetables and Fruits. J. Food Sci. Technol..

[29] Zumbado-Chinchilla C., Arroyo-Esquivel L., Cortés-Muñoz M., Incer-González A.I., Esquivel P.E. (2024). Effect of Lipid Addition on Carotenoid Bioaccessibility in a Dairy-Based Papaya (*Carica papaya*) Beverage. ACS Food Sci. Technol..

[30] Molteni C., La Motta C., Valoppi F. (2022). Improving the Bioaccessibility and Bioavailability of Carotenoids by Means of Nanostructured Delivery Systems: A Comprehensive Review. Antioxidants.

[31] Mapelli-Brahm P., Stinco C.M., Meléndez-Martínez A.J. (2018). Comparative Study of the Bioaccessibility of the Colorless Carotenoids Phytoene and Phytofluene in Powders and Pulps of Tomato: Microstructural Analysis and Effect of Addition of Sunflower *Oil*. Food Funct..

[32] Yan X., Huang J., Huang L., Luo C., Li Z., Xu P., Tan K., Cheong K.L., Tan K. (2024). Effects of Dietary Lipids on Bioaccessibility and Bioavailability of Natural Carotenoids. LWT.

[33] Morón-Ortiz Á., Karamalegkos A.A., Mapelli-Brahm P., Ezcurra M., Meléndez-Martínez A.J. (2024). Phytoene and Phytoene-Rich Microalgae Extracts Extend Lifespan in C. Elegans and Protect against Amyloid-β Toxicity in an Alzheimer’s Disease Model. Antioxidants.

[34] Perazzoli G., Luque C., León-Vaz A., Gómez-Villegas P., Rengel R., Molina-Márquez A., Morón-Ortiz Á., Mapelli-Brahm P., Prados J., Melguizo C. (2024). Preliminary Assessment of the Protective and Antitumor Effects of Several Phytoene-Containing Bacterial and Microalgal Extracts in Colorectal Cancer. Molecules.

[35] Schweiggert R.M., Steingass C.B., Heller A., Esquivel P., Carle R. (2011). Characterization of Chromoplasts and Carotenoids of Red- and Yellow-Fleshed Papaya (*Carica papaya* L.). Planta.

[36] Erdman J.W., Bierer T.L., Gugger E.T. (1993). Absorption and Transport of Carotenoids. Ann. N. Y. Acad. Sci..

[37] Van Buggenhout S., Alminger M., Lemmens L., Colle I., Knockaert G., Moelants K., Van Loey A., Hendrickx M. (2010). In Vitro Approaches to Estimate the Effect of Food Processing on Carotenoid Bioavailability Need Thorough Understanding of Process Induced Microstructural Changes. Trends Food Sci. Technol..

[38] Brodkorb A., Egger L., Alminger M., Alvito P., Assunção R., Ballance S., Bohn T., Bourlieu-Lacanal C., Boutrou R., Carrière F. (2019). INFOGEST Static in Vitro Simulation of Gastrointestinal Food Digestion. Nat. Protoc..

[39] Stinco C.M., Benítez-González A.M., Meléndez-Martínez A.J., Hernanz D., Vicario I.M. (2019). Simultaneous Determination of Dietary Isoprenoids (Carotenoids, Chlorophylls and Tocopherols) in Human Faeces by Rapid Resolution Liquid Chromatography. J. Chromatogr. A.

