# Supplementary material for: Isolation of Carrot Chromoplasts and Assessment of Their Carotenoid Content and Bioaccessibility
Source: Molecules. 2025 Mar 12;30(6):1267. doi: 10.3390/molecules30061267 (PMC11944930; doi:10.3390/molecules30061267)
Supplement: Supplementary file 1 [file molecules-30-01267-s001.zip › molecules-3409689-supplementary.pdf]

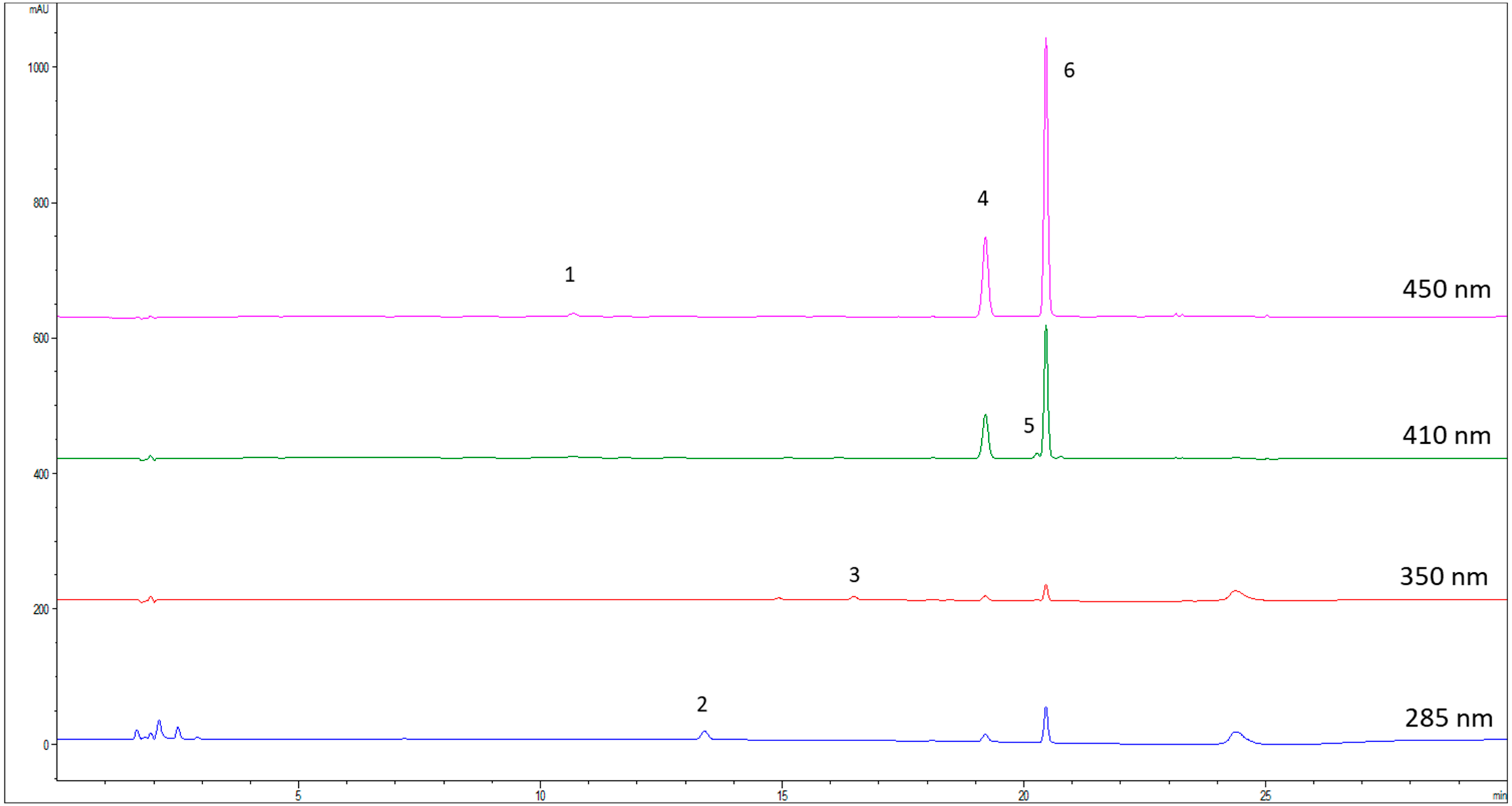

Figure S1. Chromatographic profile of carrot roots

**1: lutein, 2: phytoene, 3: phytofluene, 4: α-carotene, 5: ζ-carotene, 6: β-carotene*
